# Simulated mental imagery for robotic task planning

**DOI:** 10.3389/fnbot.2023.1218977

**Published:** 2023-08-24

**Authors:** Shijia Li, Tomas Kulvicius, Minija Tamosiunaite, Florentin Wörgötter

**Affiliations:** ^1^Third Institute of Physics and Bernstein Center for Computational Neuroscience, University of Göttingen, Göttingen, Germany; ^2^Department of Child and Adolescent Psychiatry and Psychotherapy, University Medical Center Göttingen, Göttingen, Germany; ^3^Faculty of Computer Science, Vytautas Mangnus University, Kaunas, Lithuania

**Keywords:** mental imagery, deep learning, robotic planning, artificial neural network, human-interpretable

## Abstract

Traditional AI-planning methods for task planning in robotics require a symbolically encoded domain description. While powerful in well-defined scenarios, as well as human-interpretable, setting this up requires a substantial effort. Different from this, most everyday planning tasks are solved by humans intuitively, using mental imagery of the different planning steps. Here, we suggest that the same approach can be used for robots too, in cases which require only limited execution accuracy. In the current study, we propose a novel sub-symbolic method called Simulated Mental Imagery for Planning (SiMIP), which consists of perception, simulated action, success checking, and re-planning performed on 'imagined' images. We show that it is possible to implement mental imagery-based planning in an algorithmically sound way by combining regular convolutional neural networks and generative adversarial networks. With this method, the robot acquires the capability to use the initially existing scene to generate action plans without symbolic domain descriptions, while at the same time, plans remain human-interpretable, different from deep reinforcement learning, which is an alternative sub-symbolic approach. We create a data set from real scenes for a packing problem of having to correctly place different objects into different target slots. This way efficiency and success rate of this algorithm could be quantified.

## 1. Introduction

Task planning is the process of generating an action sequence to achieve a certain goal. To do this with conventional AI-planning, one needs to rigorously define symbolic structuring elements: the planning domain include planning operators, pre- and post-conditions, as well as search/planning algorithms (Fikes and Nilsson, 1971; Hoffmann, 2001; Helmert, 2006). While this is powerful in different complex scenarios, most every-day planning tasks are solved by humans without explicit structuring elements (even without pen and paper). Modern neural network-based methods can predict the required action, given a scene, without any aforementioned symbolic pre-structuring (Hafner et al., 2020; Schrittwieser et al., 2020). However, the reasons for the decisions made by a neural networks usually remain opaque and interpretation by a human is impossible. Thus, networks elude explanations, which, however, might be important in human-robot cooperation tasks. Based on this need, we are suggesting a planning approach based on human-understandable entities: image segments, objects, and affordances, but no explicit domain descriptions.

Our new method for task planning called Simulated Mental Imagery for Planning (SiMIP) consists of the following components: perception, imagination of the action effect, success checking, and (re)planning. This is similar to everyday human plans, comprising few steps only, being many times ad hoc, involving frequent success-checking and re-planning (Hesslow, 2012). Note, however, that we abstract away from agent self-modeling (as in Kwiatkowski and Lipson, 2019) and only produce mental images of successive scenes. If one wants to extract parameters required for robotic execution, such as locations of objects to be grasped or target locations of where to put the objects, one has to post-process the mental images showing scenes before the action and after the action. In addition, we do not include actions of other agents in our mental models (as in Krichmar et al., 2019).

Extending affordance-based approaches, which analyze one scene at a time (Stark et al., 2008), we add to our architecture generative adversarial networks (GANs) for simulated imagery of scenes following an action. Given the impressive performance of GANs in realistic image generation (Karras et al., 2019; Yu et al., 2019), one could potentially use them to envision outcomes of robot manipulation. However, when handling complex scenes, GANs tend to suffer from instabilities in learning. Moreover, when processing complex scenes in an end-to-end manner, network behavior is hard to explain (e.g., see Nair and Finn, 2019). Instead, we suggest obtaining future scenes by re-combinations on an object-by-object basis, with a GAN-based “imagination” step for the completion of individual objects. This is reminiscent of object-centric approaches that address scenes in object-by-object manner in latent space, (e.g., see Veerapaneni et al., 2020; Chang et al., 2022). However, we prefer to keep the model explicit for achieving more stable training and performance.

As stated above, we use a simulated mental imagery process, which creates images of the outcome of an imagined action, then we use the imagined outcome as the input for the next imagined action, and so on. This way we can create a planning tree composed of images for which conventional search algorithms can be used to arrive at an image sequence that leads to the goal. While the tree remains sub-symbolic, due to the object-wise treatment of the imagined scenes, it can be readily post-processed into a symbolic representation required for robotic action. Stated in natural language, from the representations employed, it is possible to deduce commands such as “pick an object with label A from the table top with center coordinate (*x*_1_,*y*_1_), and diameter B cm and place it on an empty space with center coordinate (*x*_2_,*y*_2_).” This, together with the obtained image trees, makes the approach explainable to a human both in symbolic as well as in visual terms.

We demonstrate our approach on a task of box packing, where we created and labeled a small data set for that. As we keep the neural architectures simple (the aforementioned object-by-object attitude), comparatively small data sets suffice for training. Thus, one could also address a new task by preparing and labeling a new data at limited costs. A large domain of problems including packing, stacking, and ordering (of table-tops, shelves) can be addressed this way.

The article is structured as follows. In section II, we discuss related work. Subsequently, an overview of our approach is presented in section III and implementation details are described in section IV. In Section V, we present experiments and results, and, finally, in section VI, we provide a conclusion and outlook.

## 2. Related work

We will first briefly discuss classical symbolic and then neural network-based sub-symbolic planning. We discuss the usage of physical simulation in planning in respect to mental imagery of future scenes used in our study. Then we provide an overview of affordance recognition, focusing on aspects relevant to our framework. In the end, we briefly review the usage of neuro-symbolic representations in visual reasoning, which is also to some degree related to our approach.

### 2.1. Symbolic planning

Classical planning techniques originating from STRIPS (Fikes and Nilsson, 1971) are the usual choice for decision-making for robotic execution. They use a symbolic, logic-based notation compatible with human language that permits an intuitive domain specification (Ingrand and Ghallab, 2017). Contemporary planning techniques go a step forward and handle real-world uncertainties using probabilistic approaches (Lang and Toussaint, 2010; Kolobov, 2012; Zhang et al., 2017). Despite the recent progress of such planning applied to robotics, these techniques are still subject to the symbolization problem mentioned before: all the relevant aspects for the successful execution of the robotic actions should be considered in the planning problem definition using scenario-specific domain descriptions.

To reduce hand-crafting, learning methods have been designed for aiding the domain definitions (Ugur and Piater, 2015; Asai and Fukunaga, 2018; Konidaris et al., 2018; Ahmetoglu et al., 2022). However, learning is not effort-free as data sets of pairs of pre- and post- conditions are required. In case of classical techniques, many constraints and problem pre-structuring is needed (Konidaris et al., 2018). In case of deep learning approaches, most often latent space representations are used for obtaining “symbols”. Experimentation of how many symbols (i.e., latent variables) does one need is required (Ahmetoglu et al., 2022), while any human-understandable meaning of these symbols can only be hand-assigned post hoc. Thus, symbolic representation learning, though possible, requires quite some additional design efforts. Generalization of the developed representations many times requires additional machinery, where objects and effects need to be assigned into classes, based on similarities in some feature space (Ugur and Piater, 2015; James et al., 2022), where the feature space is used for generalization afterward. Thus, though promising, learning of planning operators remains relatively complex and, thus, is not frequently used in practice.

### 2.2. Simulation

Physical simulation is another way for future state prediction and simulation-based approaches for planning also exist. The fusion of simulation of sensing and robot control in virtual environments is an important development leading to the application of such techniques in robotics (Rossmann et al., 2012). Planning of actions based on simulations has been done both in the realm of classical (Kunze et al., 2011; Bozcuoglu and Beetz, 2017) as well as deep-learning (Hafner et al., 2019) methods. To perform simulations, however, one needs robot- and object-models as well as a full specification of the scenario. In industrial tasks, CAD models of parts and setups are usually available. However, this is usually not the case in everyday environments. In this study, we are not concerned with industrial, high-precision robotic actions, but we are targeting the everyday domain. Most actions need only to be “fairly” accurate there, and, thus, one is not forced to simulate actions and their outcomes with the highest precision. Our method, thus, exploits mental simulation in the form of imagination of future scenes instead of physical simulation.

### 2.3. Sub-symbolic planning using neural networks

Deep reinforcement learning approaches allow learning action selection strategies in complicated worlds. Here, explicit symbolic representations are not required as actions can be deduced from the learned value function given the current scene (e.g., see Schrittwieser et al., 2020, but see also more citations below). Such models are then capable of predicting future states, either at some level of abstraction, e.g., hidden/latent variables (Racanière et al., 2017; Hafner et al., 2020), or as complete images (Ha and Schmidhuber, 2018; Kim et al., 2020). Predicting future states helps training the models as this way hypothetical future developments can be obtained. However, reinforcement learning requires probing very many consecutive states. Thus, such approaches, as for now, have been mainly developed for computer games, where there are easy ways to register state-action sequences. When using imitation learning, which reduces data requirements, 3D simulated environments as well as real scenes can be addressed (Xu et al., 2019; Lin et al., 2023). Task and motion planning problem can be formulated and learned similar to reinforcement learning approaches (Driess et al., 2020). Stereotyped tasks can be attained in real-world experiments through long self-supervised experimentation by a robot (Ebert et al., 2018), where this can be unavailable or too expensive for developing concrete applications. Different from all that, our approach does not require action sequences or pre- and post- condition pairs. Conventional approaches suffice here for learning of the following entities, which we need: object detection, object completion, and affordance segmentation in the scene. These allow performing planning for us.

### 2.4. Affordance recognition

The term “affordance” originates from cognitive psychology (Gibson, 2014). The set of affordances can be briefly described as the set of momentarily possible interactions between an agent and its environment. In robotics, this term very often takes the meaning of “which actions could a robot perform in a given situation (with some given objects)?” The goal of affordance segmentation is to assign probabilities for a set of affordances to every location in an image. A straightforward problem is trying to estimate affordances of whole objects (Stark et al., 2008; Zhu et al., 2014). However, affordances can also be detected for multiple objects in the scene (Do et al., 2018; Lüddecke et al., 2019). Studies exist predicting affordances resulting *after* an action has been executed, aiding planning (Xu et al., 2021). Alternatively, in this study, we will obtain future affordances through imagination of future scenes, thus pixel-wise affordance segmentation of scenes is enough for us.

### 2.5. Neuro-symbolic representations

Related to planning are visual reasoning tasks, such as visual question answering (VQA) (Suarez et al., 2018; Yi et al., 2018; Mao et al., 2019) which works through employing symbolic reasoning on images. These methods, similar to ours, include scene parsing modules; however, in addition, they heavily rely on NLP modules. We do not need NLP modules as our aim is individual object manipulation, where object specificity beyond its affordances is not considered. Related to our approach, we have video de-rendering tasks, where a latent representation is pushed toward an interpretable structure, by including a graphics engine into the decoder (Wu et al., 2017). Other elaborate mechanisms exist to obtain symbolically meaningful latent representations (Veerapaneni et al., 2020). We, however, do not go into the direction of interpretable latent representations but rely on explicitly modeling individual objects in the scene as instances with affordances. Finally, graph neural networks may be applied for planning tasks, where geometric and symbolic information of a scene is supplied to the algorithm (Zhu et al., 2021). Different from all mentioned algorithms here, we avoid complicated network structures in order to avoid heavy demands on the amount of data required for training. We also avoid task pre-structured architectures, so that the application of the algorithm in a new situations is made easy.

## 3. Overview

**Figure 1:**
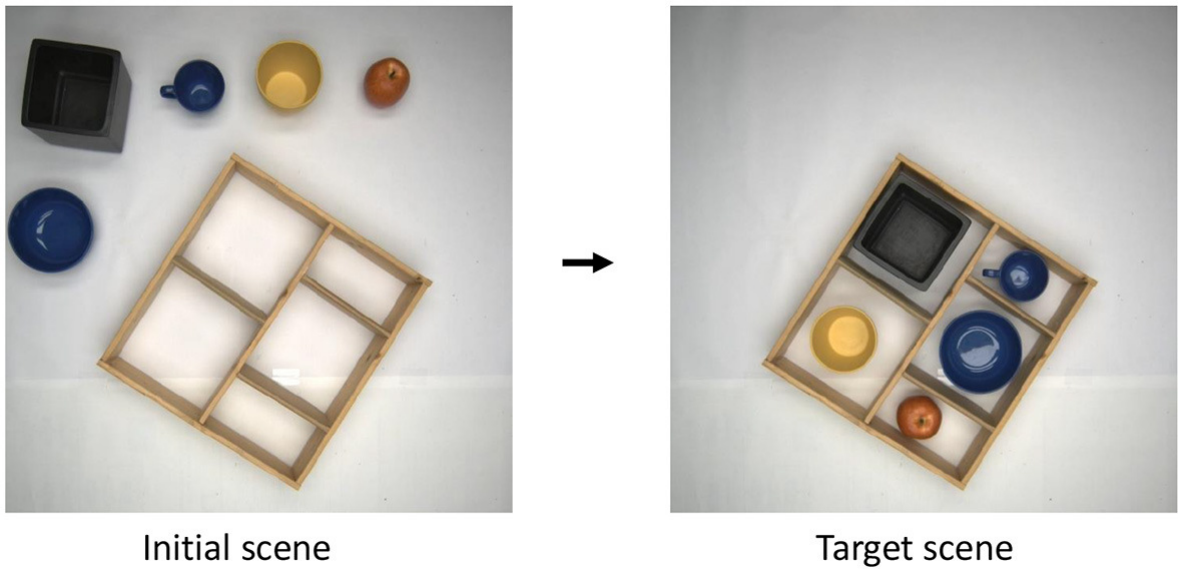
Task definition. The table top has to be ordered by putting all objects in the given box. The target is to leave no objects outside the box. Note the “target scene” here is presented only for illustration purposes as all other configurations, where there is no object left outside the box, would be considered valid too.

We are solving the task of ordering a desktop, where the system is presented with an initial scene (see Figure 1), and the goal is to put the objects in the provided box, so that there are no objects remaining outside of the box. Thus, the algorithm is not provided with the target scene as such but only with the condition that the table-top outside the box has to be free. The box can be initially empty, as shown in the figure, or partially filled. Initial filling of the box may be incorrect, with too small objects occupying compartments required for putting in a bigger object. Furthermore, an initial scene with no objects outside of the box is also a valid scene, where we expect the answer from our algorithm such that nothing needs be done.

**Figure 2:**
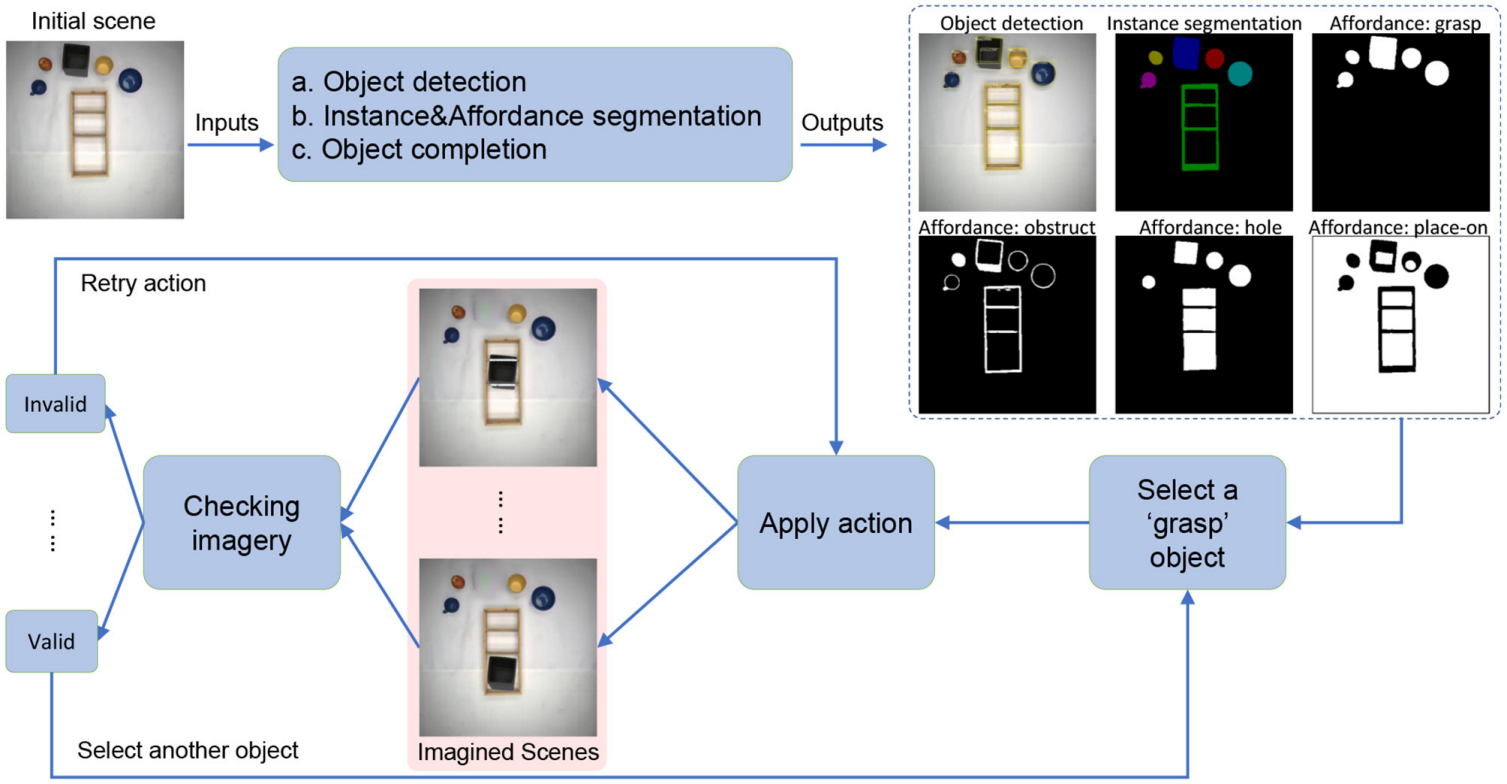
Flow diagram of our approach. Our system contains two main parts: scene understanding and action planning. For scene understanding, we use three deep networks, (a) object detection, (b) affordance and semantic segmentation, and (c) object completion. The details of the training and inference process can be seen in Figure 3. Through the scene understanding part, we can get the complete shape of the background and each individual object and its affordance class. Then, we can apply actions such as move and rotate to the object and use the information obtained from the affordance map to check whether the action is valid or not. If it is valid, we can perform the next action.

In Figure 2, the general workflow of our system is visualized, which we will describe next (for more details, see next section). We take as input an initial scene. First, we perform object detection and pixel-level instance (object) segmentation. In addition to this, we also create an affordance map for the initial scene, which assigns to the scene pixel-level affordances. We then perform object completion (de-occlusion) using a generative adversarial network (GAN). This allows us to split the whole scene into background and a set of complete individual objects. This is followed by pose estimation (not shown in the flow diagram).

Following that, we imaginarily-execute actions (i.e., generate post-action images), where we can choose from pick and place, rotate, or flip vertically. After a post-action image was generated, we perform a validity checking process determining if an imagined action has yielded a permissive result.

**Figure 3:**
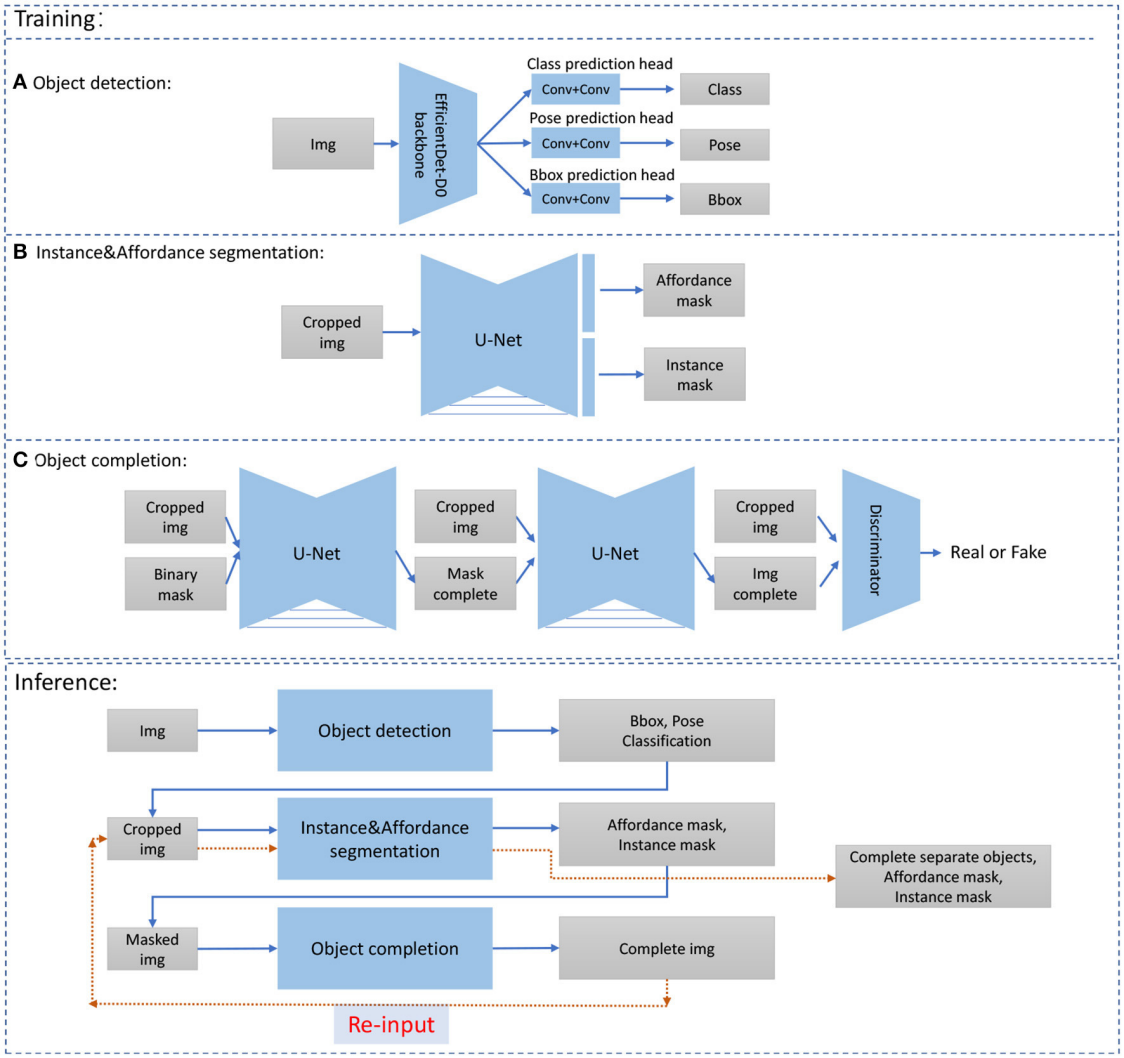
Training and inference of our model. In training, we have **(A)** object detection, **(B)** Instance& Affordance segmentation, and **(C)** object completion (de-occlusion). In the training phase, we train the three models individually and then combine the obtained results in the inference phase. Note that after finishing, the object completion [**(C)**, above], we need to do affordance segmentation [**(B)**, above) again, to get the complete object corresponding to the affordance classes (see red arrows). Bbox=bounding box. Details are explained in subsection 4.2.

Thus, in summary, we have the workflow: (a) object detection, (b) instance and affordance segmentation, (c) object completion, (d) pose estimation, (e) application of the action, and (f) evaluation of the result. Parts (a), (b), and (c) require deep network models, where the architectures for training and inference are given in Figure 3. Details of implementation will be given in Subsection 4.2 below.

**Figure 4:**
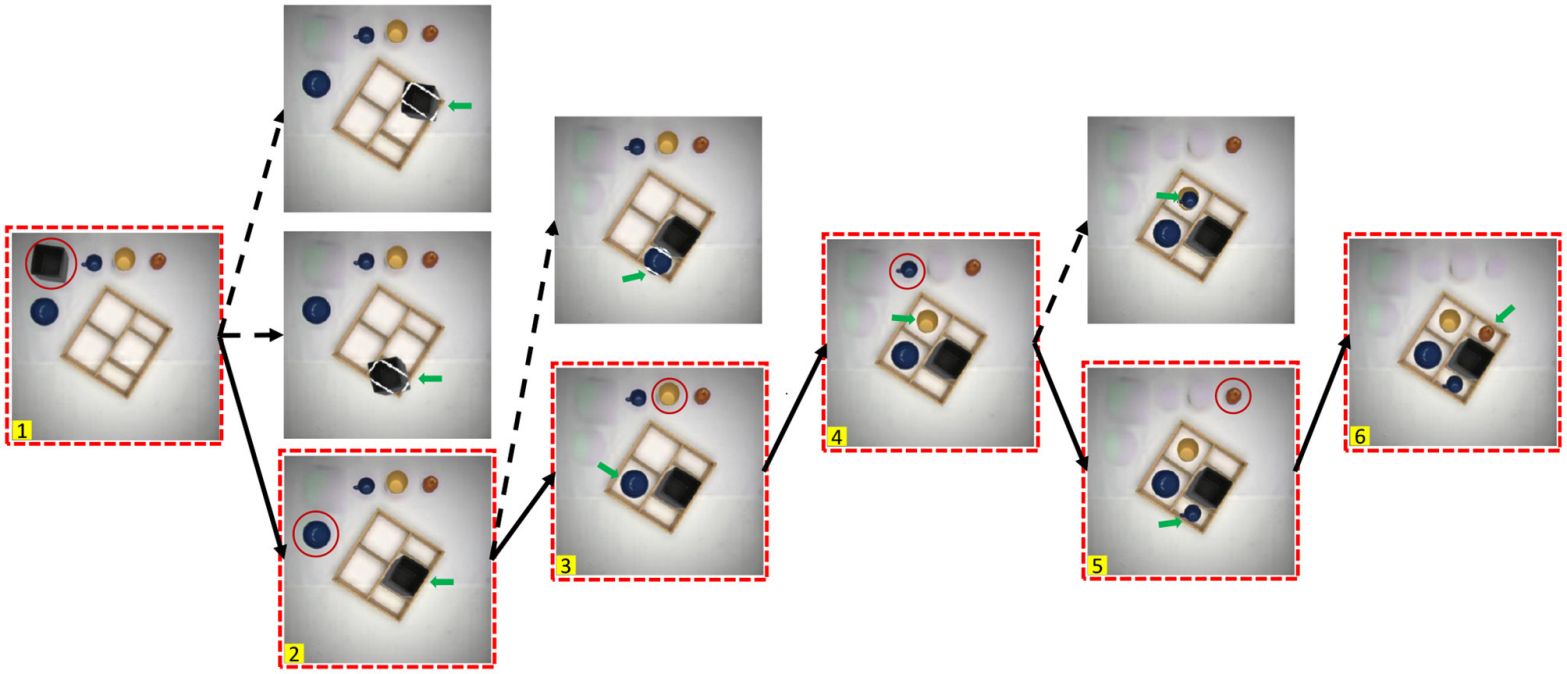
Demonstration of a planning tree. Each column represents an action step, the branches represent possible actions, and each action is based on an imagined scene, where the previous action had been completed. The red dashed boxes mark the scenes indicating the valid planning sequence and are numbered consecutively (these numbers are used in Algorithm 1). Red circles indicate the objects on which the action is applied. The green pointer indicates where the object marked by the circle in the previous image has been placed.

*Quantification*: We use a set of initial scenarios and create decision trees based on imagined scenes (see Figure 4) and check validity of the scenes. All valid image sequences then represent valid plans, where pre- and post-conditions are implicitly encoded by the images. This way, we can quantify whether or not such a system shows degradation along several planning steps, determining different scenarios, manipulation sequences, and its actual usefulness for planning and execution. In Algorithm 1, we show in a formal way how a symbolic plan for a robot can be extracted from the image-based plan shown in Figure 4. For more details, see next section.

**Algorithm 1:** Translating a visual plan into a robotic-compatible symbolic plan with parameters. Entities and parameters used in translation are extracted alongside the imagination process.

Variables:*class_label* # determined for each object in each image, including box compartments
*object_name* # of each individual object
*image_ref* # reference to images in the valid plan sequence: image_1 to image_n
*bounding_box* # parameters of the outer edges of the object in 3D
*rotations_angle* # between the same object in two consecutive images
Functions:
**Bbox**(*object_name, image_ref* ) # Read out of a bounding box, given object name and image reference
**Grasp**(*class_label,bounding box*) # for grasping of object, given its class label and bounding box
**Place_at**(*class_label,bounding_box*) # for placing object at object of class label, given its bounding box
**Rotate**(*object_name,rotation_angle*) # for rotating of object using angle between two consecutive images
**Flip**(*object_name*) # clockwise flip an object by 90°.
Variable Assignment (for the example in Figure 4):
*class_label* = {“can”, “bowl”, “cup”, “apple”,“compartment”}
*object_name*= {“black_can”, “blue_bowl”, “yellow_cup”, “blue_cup”, “red_apple”,
“compartment_1”, “compartment_2”,…, “compartment_5”}
*image_ref* ={“image_1”,…, “image_6”}
*bounding_box*={parameters of all bounding boxes}
*rotation_angel* = {parameters of all rotation angles of all objects}
Plan (for the example in Figure 4):
**Grasp**(“can”,
**Bbox**(“black_can”, “image_1”))
**Rotate**(“black_can”,rotation_angle)
**Place_at**(“compartment”,
**Bbox**(“compartment_4”, “image_1”))
**Grasp**(“bowl”,
**Bbox**(“blue_bowl”,”image_2”))
**Place_at**(“compartment”,
**Bbox**(“compartment_2”, “image_2”))
**Grasp**(“cup”,
**Bbox**(“yellow_cup”, “image_3”))
**Place_at**(“compartment”,
**Bbox**(“compartment_1”, “image_3”))
**Grasp**(“blue_cup”,
**Bbox**(“blue_cup”, “image_4”))
**Place_at**(“compartment”,
**Bbox**(“compartment_5”, “image_4”))
**Grasp**(“apple”,
**Bbox**(“red_apple”, “image_5”))
**Place_at**(“compartment”,
**Bbox**(“compartment_3”, “image_5”))

## 4. Implementation

### 4.1. Data set

The data to train and evaluate our proposed method are created from a real environment. We used a top-view camera positioned 100 cm above the center of the table and collected images with a resolution of 1, 024×7, 68 pixels. Note that the usage of the top-view camera is not a restriction of this method. At the end of this study, we show that top views can be generated by inverse perspective mapping. Hence, similar to human imagination processes, where we employ some canonical “internal view” onto the imagined scene, here the top view serves as the canonical perspective for our planning method.

The data set includes eleven different objects from seven classes: can, cup, plate, bowl, apple, box, and cuboid, where cups and cuboids are two each, and there are three different boxes. We used the following procedure for data collection: (a) we randomly placed the box and some objects on a table; (b) we applied random actions by hand, changing position or orientation of one of the objects or the box and took a picture after each action has been accomplished. We repeated (a) and (b) multiple times. This way we collected 1196 scenes. Each scene contains at least one object with a unique pose and position. Afterward, the scenes were labeled with instance and affordance annotations. For instance annotation, the seven aforementioned object categories and four different affordance categories (grasp, place-on, obstruct, and hole; for description, see Table 1) were considered and extracted for all visible regions. It is important to note that our data set does not structure collected images into pairs: (image before the action, image after the action) and does not include all possible goal configurations.

**Table 1:** Description of the set of affordances used.

**Affordance**	**Description**
Grasp	Areas that can be grasped to apply another action.
Place-on	A surface, where objects can be placed.
Obstruct	Areas, where objects are not allowed to be put.
Hole	Hollow space in a solid object.

### 4.2. Network implementation details

Many of the approaches combined here represent standard methods, and will, thus, only be described briefly. Note that neural networks for object detection, instance and affordance segmentation, as well object completion are first trained separately using our dedicated data set. Afterward, the results from different networks are integrated to obtain the imagined planning tree, where we provide details of that integration.

For object detection, considering the size of the data set, we used EfficientDet-D0 (Tan et al., 2020) as the backbone network. During training, we applied horizontal flipping, scale jittering, and random masking to perform data augmentation, and then we resized the images to 512×512 pixels as the input. We modified the output and added a pose classification head to predict whether the object is placed vertically or horizontally. The model is trained for 200 epochs with a total batch size of 4. We also used the SGD optimizer with momentum 0.9 and reduced the initial learning rate 0.001 by factor 0.1 when the total loss has stopped improving after 3 epochs. The other parameters are same as in Tan et al. (2020) and the original loss functions are utilized.

For Instance&Affordance segmentation, we used a U-net like architecture (Ronneberger et al., 2015) and apply the same loss fuction as in Lüddecke et al. (2019). Let *C*_*s*_*k* denote the Convolution-BatchNorm-ReLU, *DC*_*s*_*k* denote double convolution layers (Convolution-BatchNorm-ReLU-Convolution-BatchNorm-ReLU), *PC*_*s*_*k* denote partial convolution layers (Liu et al., 2018) (PartialConvolution-BatchNorm-ReLU), k be number of filters, and subscript s be stride. Furthermore, *UP* denotes upsampling layers and *DN* denotes downsampling layers. Then, the encoder is defined in the following way: *DC*_1_32−*DN*−*DC*_1_64−*DN*−*DC*_1_128−*DN*−*DC*_1_256−*DN*−*DC*_1_256and the decoder by is defined by *UP*−*DC*_1_512−*UP*−*DC*_1_256−*UP*−*DC*_1_128−*UP*−*DC*_1_64. After the last decoder layer, two classification heads are applied to obtain four-dimensional output for affordance segmentation and two-dimensional output for semantic segmentation (background and main body). To work with the image with completed objects and for obtaining of the secondary segmentation mask, we cropped the image according to its axis-aligned bounding box and resized it to 256×256 pixels. Combining all the outputs of the bounding box patches, we got the Instance&Affordance segmentation for the original image. The model is trained for 400 epochs with the Adam optimizer and a learning rate 0.0001 with batch size 8. The binary cross-entropy loss is employed for classification.

For object completion, we applied two U-net-like architecture models PCNet-M and PCNet-C as in Zhan et al. (2020) as mask and image generator. For PCNet-M, we used the same structure as used in segmentation, and for PCNet-C, we used six down-sampling layers encoder (PC_2_64-PC_2_128-PC_2_256-PC_2_512-PC_2_512-PC_2_512) and six up-sampling layers decoder (PC_1_1024-UP-PC_1_1024-UP-PC_1_768-UP-PC_1_384-UP-PC_1_92-UP-PC_1_67-UP). The last layer for the decoder has no BatchNorm and ReLU. For the discriminator, an SN-PatchGAN (Yu et al., 2019) is applied which uses four convolutional layers with spectral normalization (C_2_64-C_2_128-C_2_256-C_2_512) and one convolution map to create a one-dimensional 70 × 70 output. As in Zhan et al. (2020), we also cropped each object according to its bounding box. The other parameters are same, and the original loss functions are utilized. During training, the PCNet-M and PCNet-C are trained for 200 epochs and 800 epochs, respectively, with the Adam optimizer with learning rate 0.0001 and batch size 8.

### 4.3. Pose estimation

**Figure 5:**
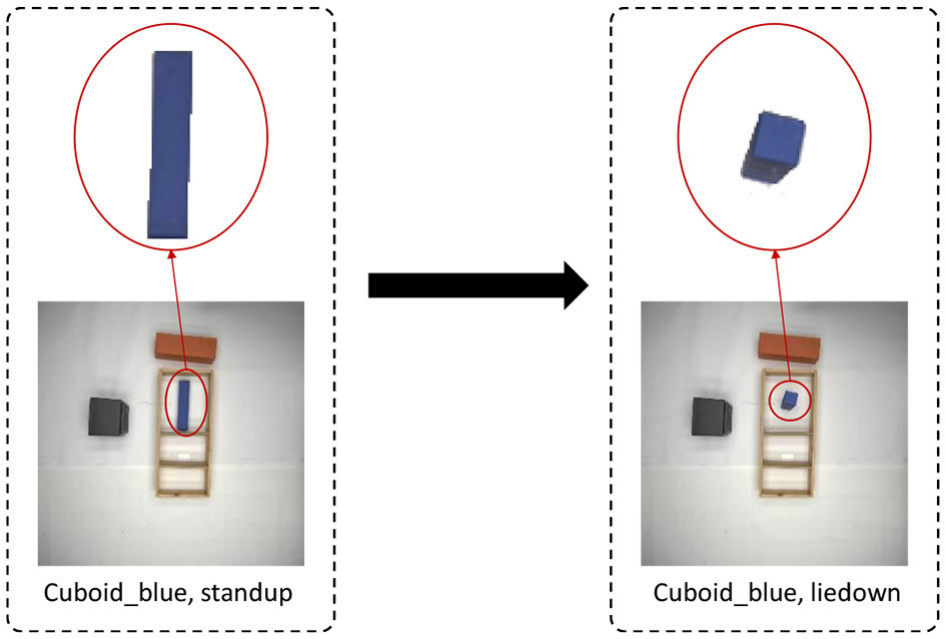
Example for pose mapping. We create a dictionary to store the horizontal and vertical pose of the blue cuboid. When we apply a flipping action on this object, we can lookup the dictionary and retrieve the corresponding pose.

The data collected in our experiments come from a top-down view of the RGB-camera image, which is appropriate for handling object movement and rotation in the horizontal direction. However, we also allow flipping of an object; for example, as shown in Figure 5, cuboid will appear different when placed horizontally or vertically. Hence, we need to predict the pose of the object, horizontal or vertical, together with object detection. Since each unique object in the experiment belongs to one category, we create a dictionary to store the horizontal and vertical poses of each object. The category and pose of the object are jointly used as primary keys, and the corresponding object's RGB image, instance segmentation map, and affordance map are saved as values. The horizontal or vertical pose of each category of objects is saved only once. When we need to flip an object, we can use this dictionary to get the flipped pose of the corresponding object.

### 4.4. Applying the action

The actions, we apply, are pick and place, rotate, and flip vertically. For pick and place to be performed, the object has to have an affordance *grasp* and the place where the object is placed shall have an affordance *place-on* or affordance *hole*; however, the *obstruct* affordance shall be not present for those pixels. In the first instance, we do not check if the object is fitting on the area of affordance *place-on* or *hole* correctly, but see the next subsection “action validation,” where we solve this. For the action rotate, the object shall have affordance *grasp*. Rotation is being performed in 15 deg. steps. For the action flip vertically, the object shall have affordance *grasp*, and the imagined action is performed by retrieving entries from the dictionary of horizontal vs. vertical poses, as was described above. The result of an imagined action is a post-action image.

To obtain the post-action image, we regarded each object as a separate layer and then we used traditional image processing methods, such as cut-and-paste and rotation, to perform the movement and horizontal rotation of the object. We take the center of the object's bounding box as the origin when applying the action. For flipping objects, we need to replace the corresponding object layer with the flipped pose according to the dictionary. The object layers are afterward overlaid on a background layer to get the resulting image showing the result of applying the action.

### 4.5. Action validation

In the last step, having obtained images after action imagination, we check whether the action is valid or not. We require that the object is not placed in an area where the affordance is “obstruct.” Thus, the checking process is based on the affordance map. For this, we define conflict areas as the intersection of the “obstruct” affordance with the manipulated object. We count the intersection pixels, where we set the threshold to 30 pixels. If the conflict area is less than 30 pixels, then we assume that the action is correct.

### 4.6. Formation of a planning tree

For the planning tree, we used a basic greedy search approach (depth-first-search) to generate a valid plan. For the initial scene, we first randomly selected an object with a picking affordance from the set of objects standing outside the box. Then, we attempted to position the object on a randomly selected placing affordance. For that, we performed a series of imaginary actions, including rotation and flipping and verified the image after each action until the object passes a validity check, which a conflict area of no more tan 30 pixels as described above. If success was not achieved by rotating the object in 15° steps either flipped or non-flipped, we proceeded to choose another object from the ones standing outside the box. If an object was successfully placed, we advance to the next planning step based on the image generated in the first planning step. Affordance-supported stacking here is also allowed. We terminate the process when there are no more objects outside, or no action exists that passes the validity check.

### 4.7. Parsing of symbolic entities

In Algorithm 1, we show an example of parsing of the valid visual plan, represented as an image sequence in Figure 4, into symbolic planning entities with parameters required for robotic execution. Note that entities used in the parsing process: class labels, bounding boxes, and the manipulated object sequence are directly obtained from the imagination process. Hence, for making a symbolic plan, it remains to collect those entities from the images and pass them to the corresponding robot action primitives. We provide the plan in an unwrapped form (instead of an algorithmic loop) to depict the full sequence of steps corresponding to the visual plan given in Figure 4. Note that this plan could be further processed (translated) into human readable sentences (not shown here) or—as an alternative—one could use automatic, neural network-based methods (see e.g., Dess̀ı et al., 2023) for image captioning to arrive also at a language description of the images. However, the latter is much more demanding than the former due to the fact that our system already provides many relevant entities and variables for sentence generation.

## 5. Experiments and results

As defined above, our task is defined as the need to organize a table top by packing objects into a box so that the table outside the box is empty. The box has differently sized partitions and, similarly, objects have different sizes and shapes.

First, we evaluated different system components: object detection, instance segmentation, affordance segmentation, and object completion. Afterwards, we evaluated the method as a whole, including ablation analysis.

### 5.1. Evaluation of the system's components

**Figure 6:**
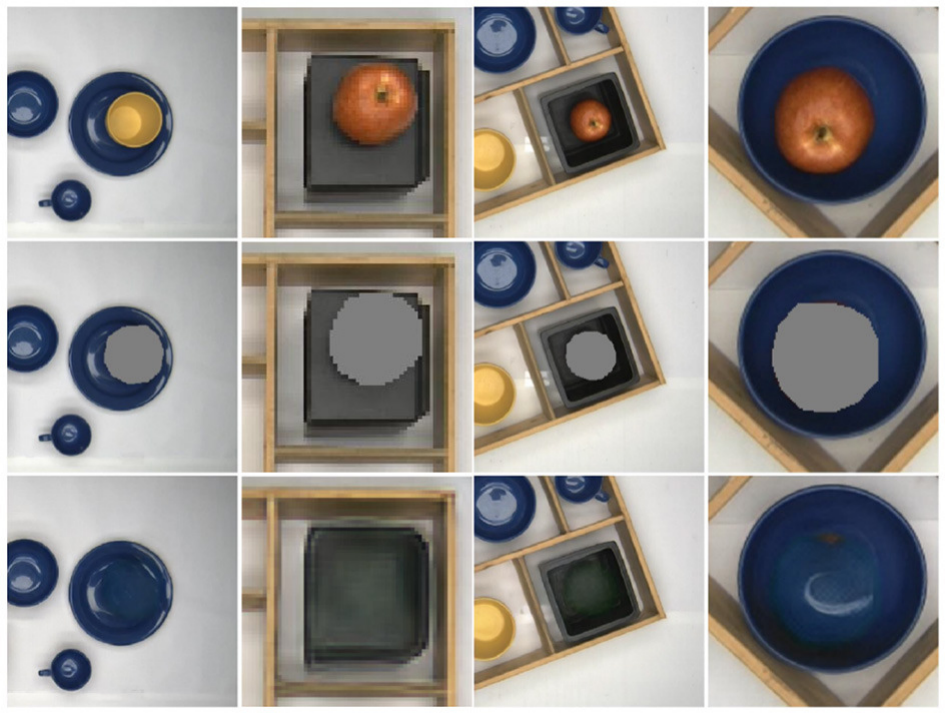
Object completion: qualitative examples. First row: image fragments from the test data set. Second row: mask of the obstructing object detected. Third row: completed object re-inserted into the scene.

We evaluated the deep learning models used in our process on the test data set, and the results are shown in Table 2. Note that for the object completion task, we need to fill-in the occluded parts of objects, where we obtain small average losses L1 and L2 ( 0.0275 and 0.0028, respectively). As we are addressing the problem using a top-view, completion mostly addresses object stacks; however, some small occlusions, occurring in case objects stand close together not directly under the camera, need this type of handling, too. Since our data set is relatively small and the difference in object appearance between the training and test sets is not significant, these deep learning models perform well in our assigned task (see Figure 6). Hence, these models build a solid foundation for the following task planning.

**Table 2:** Results for model components a, b, and c (see Figure 3 first blue box on top).

**model**	**results: mean (SD)**
Object detection: EfficientDet-D0	mAP@0.5:0.95: 71.48% (0.59%)
Instance segmentation: Unet	mIoU: 94.18% (0.36%)
Affordance segmentation: Unet	mIoU: 91.70% (0.89%)
Object completion: PCNet-M&PCNet-C	L1: 0.0275 (8.37E-05) L2: 0.0028 (5.48E-05)

mAP, mean average precision; mIoU, mean intersection over union. L1 is the L1 norm; given as mean (SD) in 5-fold cross-validation.

### 5.2. Evaluation of the method

We verified our method using 5-fold cross-validation. Scenes in the data sets differ in the number and location of objects. Our target is to place as many objects from outside the box as possible into the appropriate compartments in the box. To save computational resources, a depth-first search is used to find the complete plans, which are then checked whether they are valid. As many valid plans exist, it is costly to construct a ground-truth set for verification of validity (e.g., consider the need to account for all combinations of packing, including stacks of objects). Hence, we evaluated the obtained plans by eye.

Of the 240 test cases in each test set, there exist plans with zero up to seven packing steps. A “0 step” case corresponds to the situation, where the box is fully packed and no planning steps are required. This is included to test the system's capability to recognize also such situations. Table 3 shows how many of these different cases had been successful. The grand average success rate across all cases was 90.92%. As expected, the success rate deteriorates by 10-15% for longer planning sequences, as both imagination and planning errors accumulate.

**Table 3:** Success rates for planning cases with different plan lengths using a 5-fold cross-validation.

	**number of steps in plans**	**0 step**	**1 step**	**2 steps**	**3 steps**	**4 steps**	**5 steps**	**6 steps**	**7 steps**	**Total**
[-2ex]Our method	Total cases	28.6(3.44)	56.2(6.94)	34(2.83)	47.4(2.97)	39.2(4.97)	19.8(5.02)	7.2(1.3)	7.6(0.89)	240(0)
		Success rate	**94.47%**(1.60%)	**96.16%**(1.24%)	**89.39%**(1.63%)	**93.22%**(1.14%)	**83.86%**(4.47%)	**91.85%**(1.93%)	**74.84%**(6.31%)	**79.13%**(6.42%)	**90.92%**(1.19%)
Our method Without object completion	Total cases	26.6(3.65)	60.6 (6.27)	32.4(3.71)	45.2(2.49)	40.2(6.65)	21 (4.06)	7.4(1.14)	6.6(2.19)	240(0)
	Success rate	87.57%(4.38%)	95.01%(2.15%)	80.93%(2.73%)	89.77%(2.82%)	77.25%(3.26%)	91.52%(0.86%)	68.02%(7.57%)	52.06%(10.49%)	86.00%(0.96%)
Baseline	Total cases	–	64.6(2.97)	38.2(3.27)	47.6(4.1)	30.2(2.05)	38.6(3.21)	6.4(1.95)	0.6(0.55)	240(0)
	Success rate	–	51.01%(3.43%)	18.98%(3.51%)	3.42%(1.32%)	3.32%(0.23%)	2.60%(0.21%)	0.00%(0.00%)	0.00%(0.00%)	24.00%(2.01%)

Number of steps in plans denote factual number of steps in plans achieved by different methods. The results are reported as mean (SD). Best result in each column is emphasized in bold.

In Table 4, we analyze all cases in a step-wise manner asking whether a plan step *n* has been successful or not. On average, 623 steps were performed across all 240 test cases in the plan-search process. The overall average success rate of one step is 97.03%. Success rate deteriorates step-wise; however, only by a couple percent from step 1 to step 7. This demonstrates that the imagination process used in our study degrades the images only minimally.

**Table 4:** Success rates for step by step analysis.

	**n-th step of plans**	**step 1**	**step 2**	**step 3**	**step 4**	**step 5**	**step 6**	**step 7**	**total**
[-2ex]Our method	Total steps	213(3.61)	155.8(9.73)	120.8(11.08)	75.8(9.34)	34.8(4.97)	14.8(1.3)	8.4(0.89)	623.4(38.64)
		Success rate	**97.84%**(0.43%)	**97.17%**(0.58%)	**97.31%**(0.64%)	**95.33%**(3.05%)	**95.45%**(1.24%)	**95.99%**(3.68%)	**93.00%**(6.47%)	**97.03%**(0.77%)
Our method Without object completion	Total steps	215(2.92)	152.8(7.22)	120.4(10.21)	75.2(8.79)	35(4.24)	14(2.35)	6.6(2.19)	619(29.33)
	Success rate	95.90%(0.86%)	94.08%(1.12%)	97.10%(1.24%)	93.56%(2.59%)	92.07%(1.79%)	88.64%(3.07%)	62.86%(16.63%)	94.71%(0.76%)
[-2ex]Baseline	Total steps	226.2(1.79)	161.6(3.85)	123.4(4.39)	75.8(6.22)	45.6(4.98)	7(2)	0.6(0.55)	640.2(18.79)
		Success rate	56.60%(2.93%)	27.97%(1.80%)	19.96%(2.07%)	8.51%(1.48%)	3.00%(0.84%)	0.00%(0.00%)	0.00%(0.00%)	32.12%(1.25%)

The planning steps were performed for the testing data set using a 5-fold cross-validation. The results are reported as mean(SD). Best result in each column is emphasized in bold.

To identify the reasons of failed cases we analyzed the causes of each failure. The failures can be attributed to wrong object detection or inaccurate affordance segmentation results, which account for 45.37% and 54.63% of the failure cases, respectively. Failures due to object completion can not be evaluated directly; thus, ablation study is made on that component, as described at the end of the section.

**Figure 7:**
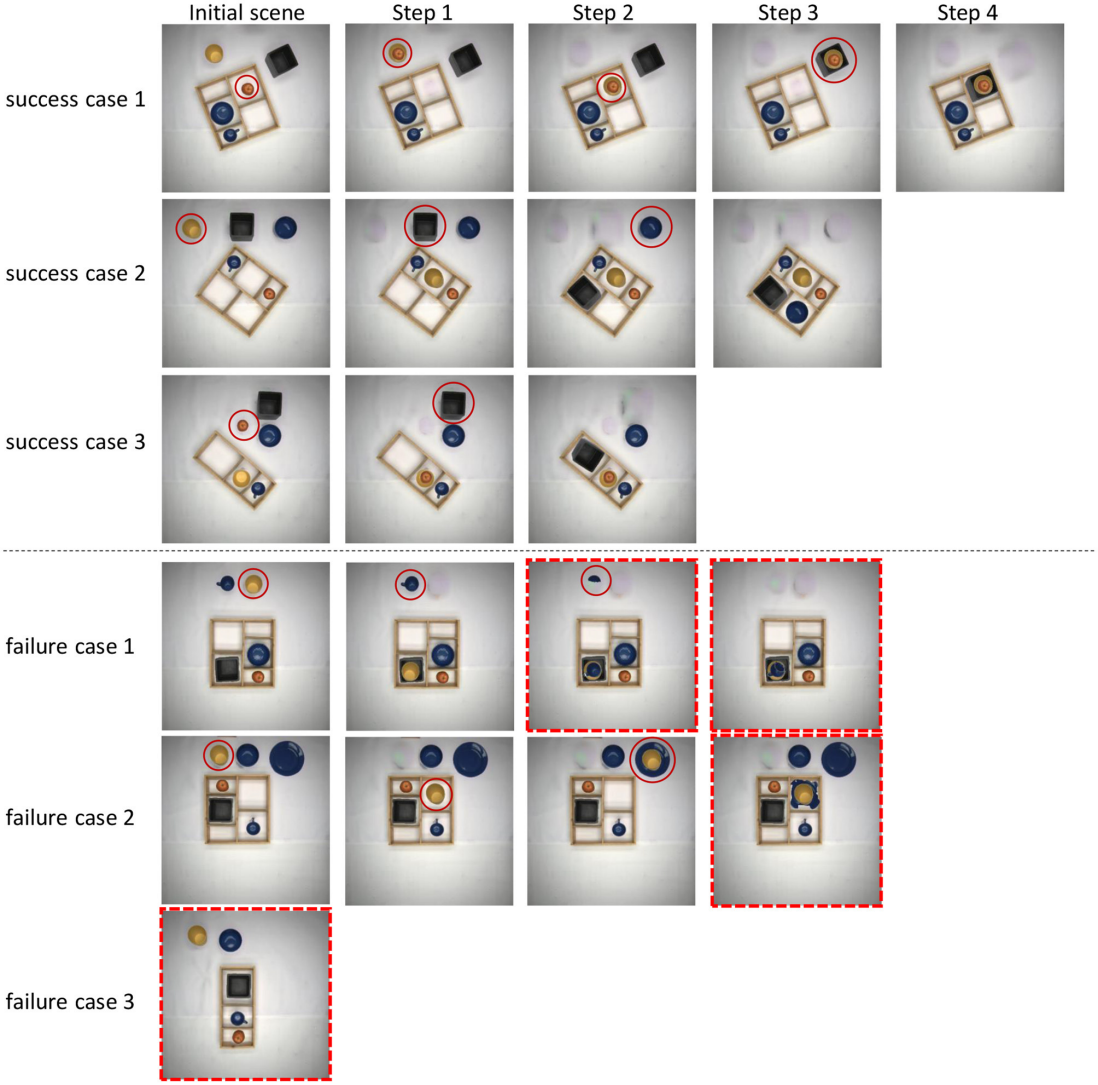
Examples of three successful and three failed plans. The first column represents the initial scene, each following column represents an individual imagined action step. Circles emphasize the objects for which the action is applied. Failed steps are marked with red dashed boxes. Explanation for individual cases: Success case 1: (1) Pick up *red apple* & place into *yellow cup*. (2) Pickup *yellow cup* (with *red apple* inside) & place into the *compartment 4* of the box. (3) Pickup the *yellow cup* (with the *red apple* inside) & place into *black can*. (4) Pickup *black can* (with *yellow cup* and *red apple* inside) & place in the *compartment 4* of the box. Success case 2: (1) Pick up *yellow cup* and place into *compartment 4*. (2) Pick up *black can*, rotate 60 deg. and place into *compartment 1* of the box. (3) Pick up *blue bowl* and place into *compartment 2* of the box. Success case 3: (1) Pick up *red apple* and place into *yellow cup*. (2) Pick up *black can*, rotate 60 deg. and place into *compartment 1*. In the failure cases, the red dashed box means an invalid step in a plan. In failure case 1, a part of the *blue cup* is incorrectly identified as another cup. The same failure cause also happens in failure case 2, where a part of the *blue plate* is identified as a can. In failure case 3, there were objects that could be packed, but no action was found in the search.

In Figure 7, we show some successful and some failed plans. Three successful plans were able to complete our box packing task. The action sequences in those plans are described in the figure legend. In the failure cases, the red dashed box means an invalid step in a plan. In failure case 1, a part of the cup is incorrectly identified as another cup, which is caused by an inaccurate result of object detection. The same failure cause also happens in failure case 2, where a part of the plate is identified as a can, which in turn leads to a wrong action. In failure case 3, there were objects that could be packed, but no action was found in the search. This is, because none of the conflict areas calculated between the “grasped” objects and all “place-on” and “hole” areas is smaller than the 30-pixel threshold value, which is caused by an inaccuracy of the result in affordance segmentation.

### 5.3. Comparison to baseline

We performed a comparison to a baseline method where we randomly choose objects and placed them on random place-affordance locations for as many times (steps), as there were objects outside the box. For each test, set the random placement was repeated four times to obtain more reliable averages. Results are shown in lines “baseline” in Tables 3, 4. Note that the baseline has a small advantage against our method as it has information how many objects there are outside the box. This leads to deterministic 100% performance in case there are no objects outside the box; t se as “not applicable” in Table 3. Otherwise, the baseline performs substantially worse than our method, which is especially visible for longer plans. Note the number of total steps in our method and in baseline method are different as the baseline method uses a simplified procedure on decision how many steps are required.

### 5.4. Ablation study

Here, we investigate the utility of different components. For the GAN-based component, the results of the study are shown in Tables 3, 4, lines “without object completion.” In all cases, the ablated version performs worse and the effect becomes especially prominent in the last steps of the plan (see the last columns of Table 4). This is expected, as with more imagination steps the need to reproduce object appearance grows. We did not make ablation study for other components of the method (e.g., object detection or affordance segmentation) as removal of those components disrupt operation of the framework completely.

As we cannot completely exclude object detection, instance and affordance segmentation from the algorithm, we made those evaluations differently. We evaluate the influence of those components on the final result by calculating success measures of components for successful and failed plans separately (Table 5). One can see that the mean average precision (mAP) for object detection is 5% smaller in failed cases, while mean intersection over union (mIoU) in instance and affordance segmentation is also a couple of percents smaller in failure as compared to success cases. This shows that there is a relation between success in the here analyzed system components and the overall system performance.

**Table 5:** Results for initial scenes of successful and failure plans.

**Model**	**Successful plans:mean (SD)**	**Failure plans:mean (SD)**
Object detection: EfficientDet-D0	mAP: 75.97% (2.06%)	mAP: 70.96% (0.19%)
Instance segmentation: Unet	mIoU: 94.13% (0.24%)	mIoU: 91.47% (1.57%)
Affordance segmentation: Unet	mIoU: 93.06% (0.10%)	mIoU: 90.68% (1.04%)

## 6. Discussion and outlook

We have presented a method for planning of packing and ordering tasks based on mental imagery, where a tree of imagined future scenes is created, and the plan is represented as a sequence of images from such a tree. Unlike methods that predict entire future images in robot manipulation scenes end-to-end (Nair and Finn, 2019), our approach involves a scene parsing process, which brings the following advantages:

- Generative processes can be supported by comparatively small data sets.
- The parsed entities can be further used for definition of robotic actions.

While successful operation of generative processes was proven in our ablation analysis, actual robotic action specification based on developed image sequences and robotic implementation will be addressed in future studies.

The approach supports explanation of the obtained plans to a human in a hybrid manner: symbolically, by using the labels of the parsed entities (see Algorithm 1) and at the sub-symbolic level by showing the human the pictures that were imagined by the system. For example, by these pictures it is easy to see what would go wrong along those planning tree branches, which were not included into the valid plan.

The developed system generalizes to different distributions of objects in the initial scenes and can achieve goal states not explicitly provided in the training data. However, the objects need to be learned for instance- and affordance segmentation as well as for generative object completion. The advantage of our method is that a relatively small data sets suffices and, thus, can be labeled with a concrete application in mind. Furthermore, due to the modularity of this system, each component within the system can be readily replaced with newly emerging state-of-the-art techniques.

**Figure 8:**
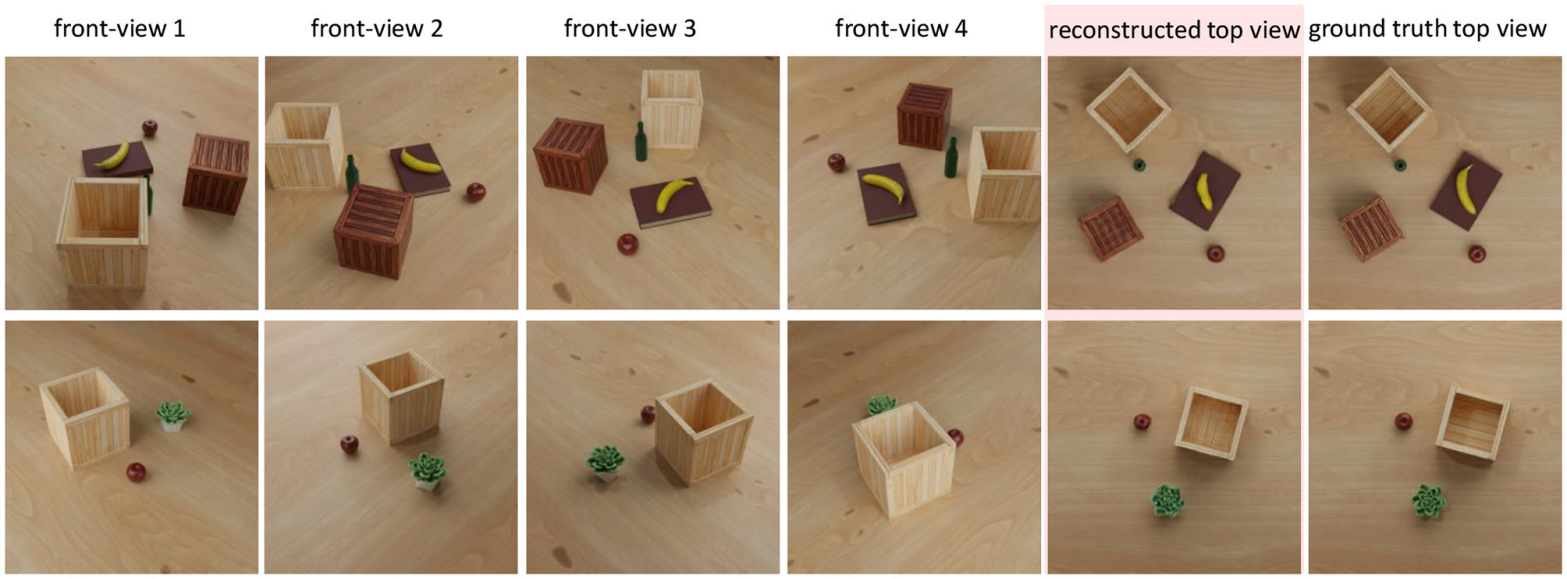
Example result of a simulated scene by applying inverse perspective mapping to create a top view from several side views.

The current algorithm uses images obtained from a top view camera. This issue does not lead to restrictions because one can recreate top view images using inverse perspective mapping methods as long as the ground plane is known. Figure 8 shows how to generate top views from different camera perspectives. Here, we created a simulated scene and placed four cameras at fixed positions around the scene for data collection. We first used inverse perspective mapping (IPM) to remap the images from four cameras into a preliminary orthographic projection based on the intrinsic and extrinsic camera parameters. Then, we used a deep network (U-net) to further correct this distorted scene and to finally get a near optimal top view image. We used 2000 images for training and 200 images for testing. As this is not in the center of this study, we directly used top view cameras, instead, to generate a canonical view for all our experiments avoiding shape deformation, which might interfere with the planning process. However, if required, IPM pre-processing can be included into our algorithms without restrictions.

Concerning the generative process introduced in our study, we performed these on an object-by-object basis and this way achieved high performance, where future frames do not substantially deteriorate over time. Though generating full images of future scenes is in principle possible and was addressed by several studies, e.g., Nair and Finn (2019), Veerapaneni et al. (2020), the obtained images are blurry (see Figure 7b in Veerapaneni et al., 2020 and Figure 1 in Nair and Finn, 2019). In some own preliminary unpublished study, we were also attempting full image generation and saw the same deficiencies too. Given that one needs anyhow individual object information for making robotic plans, applying object-by-object treatment of scenes, as now done in this study, is natural and reduces data requirements, while at the same time leading to satisfactory results.

Clearly, one cannot address very precise 3D fitting tasks for objects with complicated shapes with our approach and more specialized methods are required for that (Lin et al., 2023). For generative approaches, more advanced methods such as diffusion models (Rombach et al., 2022) can be used. In general, existing studies considering image-based foresight are mostly specialized, e.g. pouring (Wu and Chirikjian, 2020), pushing, lifting, and dragging (Ebert et al., 2018), addressing only block worlds, or rope manipulation (Wang et al., 2019), closing a door and object pushing (Nair and Finn, 2019). Here, we show that for a packing, stacking, and ordering tasks, one can simplify this by performing planning directly by visual imagination without pre/post-condition pairs for training in case of every-day accuracy requirements. In addition, from a practical perspective, it is important that for implementation of our system only deep-learning-based image analysis knowledge is needed, while domain description or reinforcement learning knowledge is not required for that.

Although our current study does not involve direct interaction with a real robot arm, for the implementation of the system on a robot, one can follow a similar approach as we did during data collection. A camera, capable of providing a top-down perspective, is required, and it needs to be set up so that the robot does not occlude the scene when in its home position. The camera has to be synchronized with the robot so that it takes an image each time after the robot has accomplished an action and has returned to the home position.

We also believe that incorporating feedback loops on a robot could enhance the success rate of the plans. We can assess the consistency between the actual scene after robot execution and the imagined scene to determine whether plan updates are necessary. If inconsistencies arise, we can choose to regenerate the plan, thereby improving the success rate of the task. For example, errors such as in Failure case 1, shown in Figure 7, where part of a cup was left behind in forward imagination could this way be corrected and would then not influence the final result. Alternatively, we can also choose to regenerate the plan after each step, which allows for continuous updates of the overall plan. This approach essentially involves making predictions for each step individually and our experimental analysis above suggests that planning only one step yields high success rates. This will be the focus of our future study.

## Data availability statement

The datasets presented in this study can be found in online repositories. The names of the repository/repositories and accession number(s) can be found below: https://doi.org/10.5281/zenodo.7904630.

## Author contributions

SL performed data set generation, developed the methods, and performed simulations and analyses. TK provided camera setup and data acquisition. MT and FW performed analyses and wrote manuscript. All authors contributed to the article and approved the submitted version.
